# Improvement of Fermentation Quality in the Fermented Total Mixed Ration with Oat Silage

**DOI:** 10.3390/microorganisms9020420

**Published:** 2021-02-18

**Authors:** Hong Yang, Bing Wang, Qing Zhang, Hui Cheng, Zhu Yu

**Affiliations:** 1 College of Grassland Science and Technology, China Agricultural University, Beijing 100193, China; hong.yang@ugent.be; 2 Laboratory for Animal Nutrition and Animal Product Quality, Department of Animal Sciences and Aquatic Ecology, Faculty of Bioscience Engineering, Ghent University, 9000 Ghent, Belgium; 3 National Key Laboratory of Animal Nutrition, College of Animal Science and Technology, Agricultural University, Beijing 100193, China; wangb@cau.edu.cn; 4 College of Forestry and Landscape Architecture, South China Agricultural University, Guangzhou 510642, China; zqing_scau@163.com; 5 Department of Civil and Environmental Engineering, Graduate School of Engineering, Tohoku University, 6-6-06 Aoba, Aramaki-Aza, Sendai, Miyagi 980-8579, Japan; chenghui@shu.edu.cn

**Keywords:** total mixed ration silage, oat, Illumina MiSeq sequencing, fermentation quality, chemical composition, *Lactobacillus* genus complex

## Abstract

The use of the fermented total mixed ration (FTMR) is a promising approach for the preservation of homogeneous feed, but changes during fermentation and links with the bacterial community of FTMR are not fully understood. This study investigated the effects of adding oat silage (OS) to the fermented total mixed ration (FTMR) in terms of fermentation, chemical composition, and the bacterial community. The fermentation quality of FTMR with 22% OS was greatly improved, as demonstrated by decreases in the butyric acid concentration, a lower lactic acid/acetic acid ratio, a larger population of lactic acid bacteria (LAB), and quicker spoilage yeast death. Further examination of the effects of various ensiling days on nutritive values showed stable crude protein and nonprotein nitrogen (NPN) contents. The concentrations of acetic acid, propionic acid, and ammonia–nitrogen (NH_3_–N) were increased following all FTMR treatments after 15 d, while the concentration of water-soluble carbohydrates (WSC) was decreased. More heterofermentative LAB, such as *Lentilactobacillus buchneri*, *Lentilactobacillus brevis*, and *Companilactobacillus versmoldensis* were found after adding 11% and 22% OS. Moreover, the addition of 22% OS caused a marked increase in both bacterial richness and diversity, dominated by the *Lactobacillus* genus complex. Among species of the *Lactobacillus* genus complex, the occurrence of *Loigolactobacillus coryniformis* was positively correlated with lactic acid, NPN, and NH_3_–N concentrations, suggesting its potential role in altering the fermentation profiles.

## 1. Introduction

Oat (*Avena sativa* L.) is a suitable cover or break forage that is used in winter rotations and has high protein and digestible fractions [1]. For these reasons, it has been extensively cultivated and planted, particularly between latitudes 35–65° N and 20–46° S [2,3], especially in Hebei, Inner Mongolia, and Gansu Province, which account for 85% of its total production in China. It is worth emphasizing that the seasonal rainfall in these areas might increase proteolysis and affect production, thereby reducing dry matter (DM) recovery [4]. In this situation, ensiling high-moisture oat with dry feed as the fermented total mixed ration (FTMR) is a potential method to solve this problem during rainfall seasons due to its many advantages, including yearly flexible processing, efficient transportation with high aerobic stability, and the provision of homogeneous feed over time for small farms [5,6]. Moreover, making FTMR with fresh oat in just one or two days is impossible when hundred acres of oat needs to be harvested at the same time. Accordingly, making FTMR with oat silage is optimal.

A hallmark feature of FTMR is the change in nutritive value due to the production of smaller metabolites in various fermentation periods [7]. Protein and soluble sugar fractions are expected to be lost during periods of prolonged storage [8], as they are transferred to some smaller molecules, like lactic acid [7]. Another characteristic of FTMR is that it contains a high concentration of microbiota that carry out metabolic functions that can aid in these nutrition modifications [9]. For example, after fermentation for 56 d, *Lentilactobacillus buchneri* and *Pediococcus acidilactici* were found to be dominant in FTMR, while no dominant bacteria were found in fresh TMR [10]. Xie et al. (2020) also stated that bacterial strains are crucial factors that influence the nutrient content of FTMR [11]. Regarding oat silage, it is dominated by the *Lactobacillus* genus complex, and *Leuconostoc* and *Clostridium* genera [12], which probably results in obvious alterations to the fermentation process. However, to date, very few studies have been carried out to investigate the changes in nutrient composition and the fermentation quality of FTMR with oat silage and links of these changes with the presence of bacterial communities. This investigation aimed to explore changes in fermentation and chemical composition and the associations of these changes with bacterial populations in FTMR during long fermentation periods.

## 2. Materials and Methods

### 2.1. Total Mixed Ration Materials and Ensiling

The TMR was designed for high-yield lactation cows (600 kg body weight, approximate milk yield of 30 kg per day), in accordance with the guidelines of the NRC (2001) [13]. The ingredient compositions of the total mixed rations are shown in Table 1. Whole oat was harvested at the heading stage from Purple Posture Company (Zhangjiakou, China). Alfalfa hay, oat hay, corn silage, and concentrates were provided by Beijing Sino Farm (Beijing, China).

As shown in Table 1, FTMR was ensiled with 0% oat silage (0% OS), 11% oat silage (11% OS), and 22% oat silage (22% OS), and to meet the nutritional requirements of high-yield lactation cows, corn silage was added. Three-hundred-and-fifty grams of TMR mixture was packed into a laboratory silo (5000 mL capacity) and sealed with a screw top and plastic tape. This was kept at room temperature (24–29 °C). The silos were opened at 0, 3, 5, 7, 10, 15, 30, and 60 days after ensiling. Each treatment was conducted in triplicate, and, in total, 81 jars (3 levels of oat silage × 8 ensiling times × 3 replicates = 72) were prepared.

### 2.2. Analysis of the Fermentation Quality

Triplicate samples of the TMRs were opened and sampled at 0, 3, 5, 7, 10, 15, 30, and 60 days after ensiling. To measure fermentation characteristics of all samples, 20 g of each silage sample was collected and homogenized in a blender with 180 mL of distilled water for 1 min and then filtered through four layers of cheesecloth [14]. The filtrate was used to measure the pH and ammonia–nitrogen (NH_3_–N), lactic acid, acetic acid, propionic acid, and butyric acid contents. The pH of the silage was measured using a glass electrode pH meter (PHS–3C, INESA Scientific Instrument, Shanghai, China). The concentration of NH_3_–N was measured using the method described by Broderick and Kang [15]. The content of organic acids was analyzed with a high-performance liquid chromatography system equipped with the Shodex RS Pak KC-811 column (Showa Denko K.K., Kawasaki, Japan), under the following analytical conditions: detector—DAD, 210 nm, SPD-20A, Shimadzu Co., Ltd., Kyoto, Japan; eluent—3 mmol L^−1^ HClO_4_, 1.0 mL min^−1^; temperature—50 °C.

### 2.3. Analysis of the Chemical Composition

TMR samples were dried at 65 °C for 48 h by oven to determine the dry matter (DM) content. The neutral detergent fiber (NDF) and acid detergent fiber (ADF) contents were determined in accordance with Van Soest et al. (1991) using heat-stable alpha amylase and sodium sulfite and are expressed as the residual ash contents [16]. The water-soluble carbohydrates (WSC) content was determined using the anthrone method described by Murphy (1958) [17]. The crude protein (CP) content was determined using method 976_05 of the Association of Official Analytical Chemists [18]. The content of nonprotein nitrogen (NPN) was analyzed using the method described by Licitra et al. (1996) [19].

### 2.4. Microbial Population Analysis by Culture-Based Method

Twenty grams of each sample was shaken with 180 mL of sterile saline solution (8.50 g/L NaCl) for around 30 min, and serial dilutions (1/10^1^ through 1/10^6^) were made in sterile saline solution. The concentration of lactic acid bacteria (LAB) was measured using the plate count method on lactobacilli MRS (de Man, Rogosa, Sharpe) agar incubated at 30 °C for 48 h under anaerobic conditions (TE-HER Hard Anaerobox, ANX-3; Hirosawa Ltd., Tokyo, Japan). Coliform bacteria counts were estimated using Violet Red Bile Agar after incubation at 30 °C for 2 days. Yeasts and molds were enumerated on spread plates of Yeast Extract Peptone Dextrose Agar and Salt Czapek Dox Agar, respectively, after incubation at 28 °C for 3–5 d. The four media were obtained from Beijing Aoboxing Bio-tech CO., Ltd., Beijing, China.

### 2.5. Microbial Diversity Analysis Based on 16S rRNA Sequencing

A total of 10 g from each triplicate FTMR sample was collected before fermentation (d 0) and at the end of the fermentation period (d 60) and stored at −80 °C for DNA extraction. Before genomic DNA extraction, a microbial pellet was obtained from each sample according to the method presented by Ni et al. (2017) [20]. Genomic DNA was extracted using the TIANamp Bacterial DNA kit (Tiangen Biotech Co., Ltd., Beijing, China) in accordance with the manufacturer’s instructions. The extracted genomic DNA was stored at −20 °C prior to bacterial 16S rRNA gene amplicon sequencing. The V3–V4 regions of the bacterial 16S ribosomal RNA gene were amplified to prepare gene libraries by PCR using the primer set 338F (ACTCCTACGGGAGGCAGCAG) and 806R (GGACTACHVGGGTWTCTAAT). Amplicons were extracted from 2% agarose gels and then quantified and purified. Purified amplicons were pooled in equimolar concentrations and paired-end sequenced (2 × 250) on an Illumina MiSeq platform by Shanghai Majorbio Bio-pharm Technology Company (Shanghai, China).

The amplicon sequencing data set was demultiplexed, and barcodes were clipped off by the sequencing service provider. Forward and reverse reads were merged, after which primer removal and quality filtering at the same sampling depth were conducted using QIIME 1 (version 1.17) in accordance with Wang et al. [21]. Operational Units (OTUs) were clustered with a similarity cut-off of 97% using Uparse (version 7.1 http://drive5.com/uparse/ access on 20 August 2015), and chimeric sequences were identified and removed using UCHIME. The taxonomy of each 16S rRNA gene sequence was analyzed by the RDP Classifier (http://rdp.cme.msu.edu/, accessed on 22 August 2015) against the silva (SSU115) 16S rRNA database using a confidence threshold of 97% [22]. The raw reads were deposited into the NCBI Sequence Read Archive (SRA) database (Accession Number: SRP198854).

### 2.6. Data Analysis

Data were analyzed by SAS 9.4 (SAS Inst. Inc., Cary, NC, USA). A mixed model was used to determine the fixed effects of time, OS proportion in FTMR, and their interaction:

Y_ij_ = µ + α_i_ + β_j_ + (α × β)_ij_ + e_ij_

where µ is the overall mean, α_i_ is time, β_j_ is the OS proportion in the FTMR, (α × β)_ij_ indicates interactions of time and the OS proportion in FTMR, and e_ij_ is the residual error.

**Figure 1:**
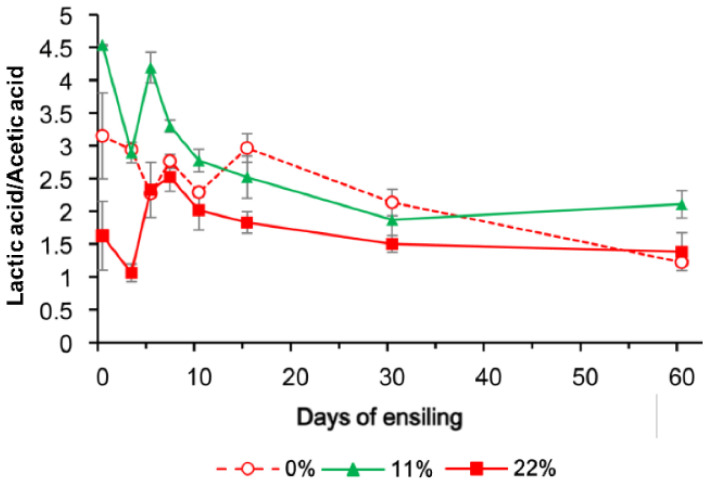
Changes in the lactic acid/acetic acid ratio in FTMR during fermentation with different oat silage content (OS) concentrations. 0%, 0% oat silage; 11%, 11% oat silage; 22%, 22% oat silage. Data were analyzed by two-way ANOVA.

**Figure 2:**
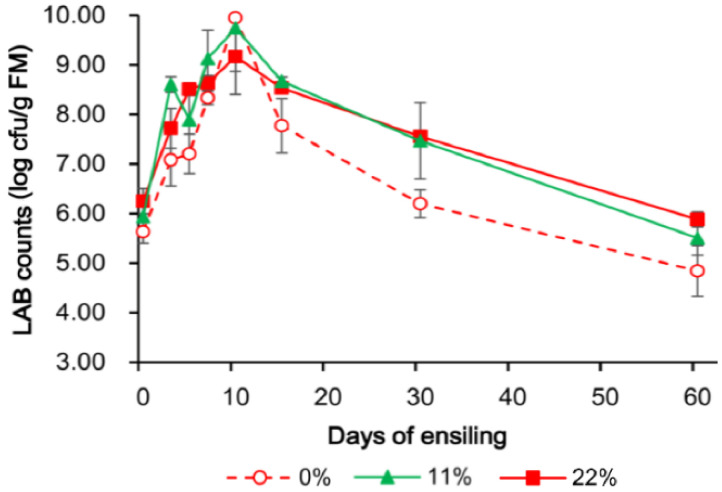
Changes in the lactic acid bacteria (LAB) count in FTMR during fermentation for different OS concentrations. FM, fresh matter. 0%, 0% oat silage; 11%, 11% oat silage; 22%, 22% oat silage. Data were analyzed by two-way ANOVA.

**Figure 3:**
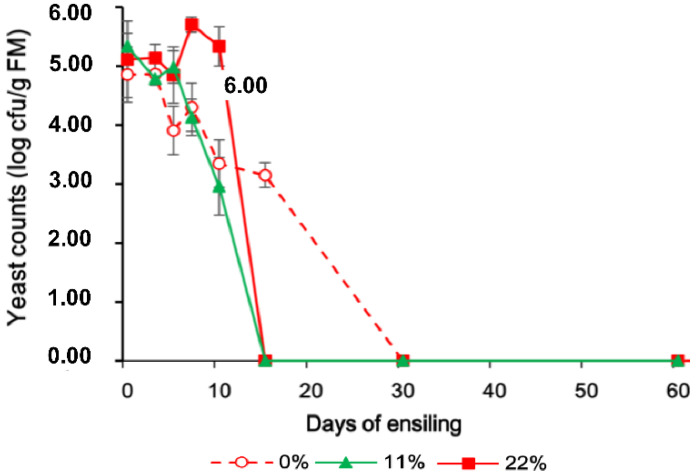
Changes in the yeast count in FTMR during fermentation for different OS concentrations. 0%, 0% oat silage; 11%, 11% oat silage; 22%, 22% oat silage. Data were analyzed by two-way ANOVA.

If there was an interaction of treatment and time, the contrasts were repeated at each time point, as shown in Figure 1, Figure 2 and Figure 3. When there was no interaction, polynomial contrasts were used to test linear and quadratic effects of increasing the fermentation time as averages over 3 OS treatments. The sequencing data were analyzed using OmicShare tools, a free online platform for data analysis (http://www.omicshare.com/tools/, accessed on 11 October 2020).

## 3. Results

### 3.1. Fermentation Quality of FTMR

The dynamic fermentation profiles of FTMR (pH, short chain fatty acid and NH_3_–N) are presented in Table 2. The pH (*p* = 0.015) and mean proportions of lactic acid (*p* = 0.016) and butyric acid (*p* = 0.014) differed among OS treatments. Compared with 0% OS, the proportions of butyric acid (days 5, 7, and 60; *p* < 0.05) and lactic acid (days 0 and 60; *p* < 0.05) were lower in samples exposed to the OS 22% treatment. The lactic acid/acetic acid ratio was the lowest in 22% OS FTMR samples before day 60 (Figure 1), which indicates that adding 22% OS enhanced heterolactic acid fermentation. The ensiling periods significantly changed other fermentation characteristics (*p* < 0.001; Table 2), but no effect on the proportion of butyric acid was found. The NH_3_–N concentration and the proportions of acetic acid and propionic acid increased in the 0% and 22% OS FTMR samples from days 15 to 60 (*p* < 0.05), while the changes were less obvious in the first 15 days. This shift in fermentation profiles was also accompanied by a decrease in pH (0%, from 5.29 to 4.49; 11%, from 5.66 to 4.66; 22%, from 5.11 to 4.57; *p* < 0.05) from days 7 to 60.

The lactate/acetate ratio, LAB content, and yeast counts are shown in Figure 1, Figure 2 and Figure 3 to indicate the interaction between OS and the fermentation period. The LAB population was greater in all OS-added treatments and had a similar change pattern, reaching a maximum concentration on day 10, followed by a modest decrease on day 60. The yeast population, however, showed a more dynamic change when more OS was added, and no yeast was detected at 100 cfu/g FM on day 15.

### 3.2. Chemical Composition of FTMR

No significant nutrient differences were found among samples exposed to the three OS treatments, except for NDF and WSC (Table 3). FTMR treated with 11% or 22% OS had a higher NDF concentration (day 5) but a lower WSC concentration (days 3, 5, and 7) when compared with those treated with 0% OS (*p* < 0.05). The number of ensiling days had a major effect on the chemical composition (*p* ≤ 0.022). After day 15, the WSC content (% DM) significantly declined to 5.18, 6.42, and 4.87 in samples treated with 0%, 11%, and 22% OS FTMR, respectively (*p* < 0.05). This was followed by a slow change until day 60. Notably, the loss of DM was less than 1% over the whole ensiling period, and this was accompanied by stable CP (on days 0 and 60) and NPN (from days 0 to 10) contents.

### 3.3. Bacterial Community and Linkages to FTMR Characteristics

**Figure 4:**
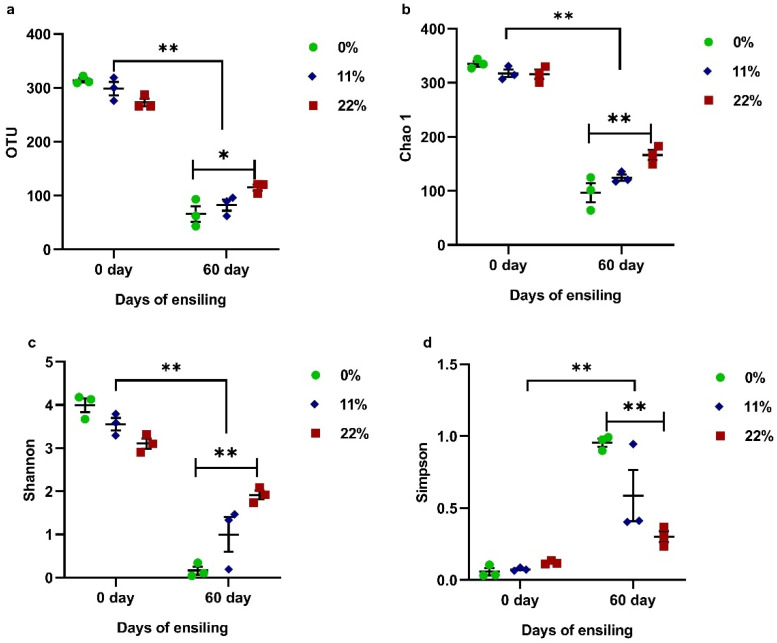
Effect of the OS content on Operational Units (OTUs) (**a**), Chao 1 (**b**), and the Shannon (**c**) and Simpson (**d**) indexes in FTMR before and after 60 days of fermentation. 0, 0% oat silage; 11, 11% oat silage; 22%, 22, oat silage. Data were analyzed by two-way ANOVA. * indicates *p* < 0.05; ** indicates *p* < 0.01.

To classify the effect of the addition of OS on microbiota, high-throughput sequencing was used to reveal changes in the bacterial community during fermentation. The alpha diversity of bacteria is shown in Figure 4. Following 60 days of ensiling, there was a marked reduction in both richness, involving OTU and Chao 1 (*p* < 0.01), and diversity, as shown by the Shannon and Simpson indexes (*p* < 0.01). Regarding the addition of OS, the most pronounced effect was found in samples treated with 22% OS, which significantly increased the richness (*p* < 0.05) and diversity (*p* < 0.01) compared with samples treated with 0% OS of FTMR.

**Figure 5:**
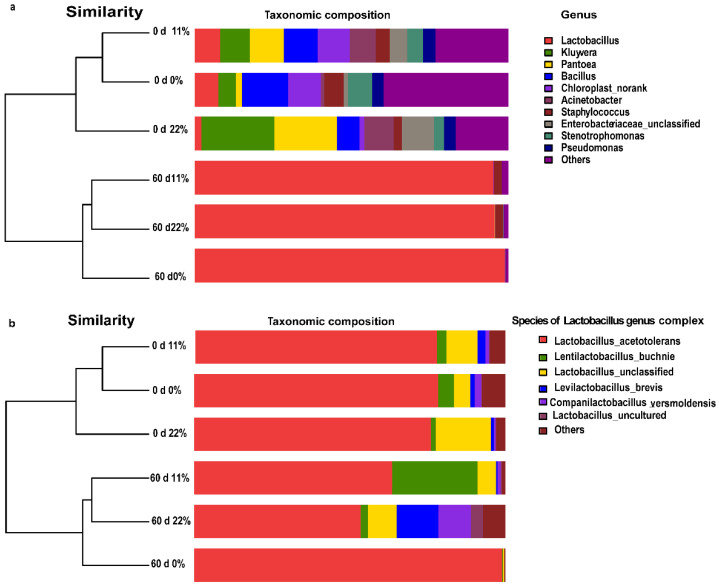
Bacterial community structure of three types of FTMR before and after 60 days of ensiling. Bacterial taxonomy at the genus level (**a**) and species taxonomy of the *Lactobacillus* genus complex (**b**). 0 d 0%, 0% oat silage on day 0; 0 d 11%, 11% oat silage on day 0; 0 d 22%, 22% oat silage on day 0. 60 d 0%, 0% oat silage on day 60; 60 d 11%, 11% oat silage on day 60; 60 d 22%, 22% oat silage on day 60.

Despite a wide diversity in the community at the genus level on day 0, *Lactobacillus*, a desirable genus complex, occupied more than 90% of the FTMR samples stored for 60 days of fermentation, regardless of treatment (Figure 5a). To better understand the bacterial structure of the FTMR samples, bacterial species of the *Lactobacillus* genus complex were identified and are shown in Figure 5b. Samples treated with 0% OS FTMR were dominated by the homofermentative *Lactobacillus acetotolerans* (99.6% relative abundance), whereas the more heterofermentative *Levilactobacillus buchneri* (20.4% relative abundance) was observed in samples treated with 11% OS FTMR. When samples treated with 22% OS were added to FTMR, more diverse heterofermentative species, such as *Levilactobacillus brevis* (17.0%), *Companilactobacillus versmoldensis* (15.2%), and *L. buchneri* (2.2%), were detected.

**Figure 6:**
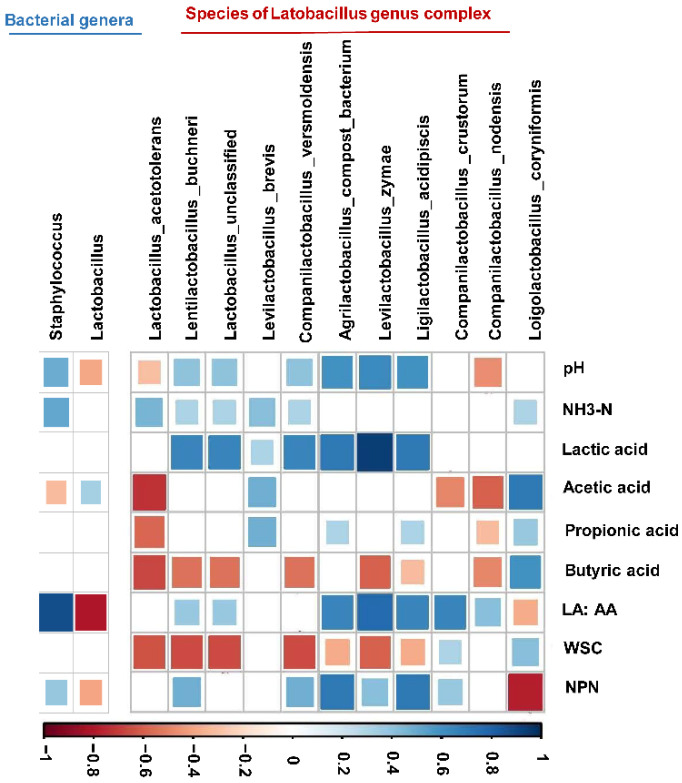
Spearman rank correlations between relative abundances of bacterial genera (>1% of community), the species taxonomy of the *Lactobacillus* genus complex, and FTMR fermentation (r ≥ 0.3). Spearman correlation coefficient values (r) range from −1 to 1. r < 0 indicates a negative correlation (blue), and r > 0 indicates a positive correlation (red). The significance of the correlation between each pair of variables is indicated by the diameters of the squares. The Spearman rank correlations results were filtered out when r < 0.3 or *p* > 0.05.

Considering the relationship between the bacteria and the fermentation products of the FTMR, Spearman correlations with an absolute correlation coefficient higher than 0.3 at both the genus and species levels were investigated. The lactic acid/acetic acid ratio showed a negative correlation with the *Lactobacillus* genus complex and a positive correlation with the *Staphylococcus* genus (Figure 6). At the species level, we found that the lactic acid and NH_3_–N concentrations were positively corelated with the occurrence of species from the *Lactobacillus* genus complex. The NPN content was positively correlated with the presence of *Ligilactobacillus acidipiscis* and *Lactobacillus* uncultured compost bacteria and positively corelated with *Loigolactobacillus coryniformis.*

## 4. Discussion

The addition of 22% OS enhanced heterolactic acid fermentation, as observed by the lowest lactic acid/acetic acid ratio in samples before day 60. When 22% OS was added into TMR, heterolactic acid fermentation became even more frequent [23]. Homofermentative LAB ensure the rapid and efficient production of lactic acid, and the pH decreases rapidly [24], while heterolactic acid fermentation allows the aerobic stability to improve [25]. Moreover, as the concentration of butyric acid, an undesirable clostridial fermentation product that could generate a loss in DM and a reduction of feed intake [26], was lower in FTMR with more oat silage. This finding further supports the idea that adding oat silage could improve the fermentation quality.

The high DM content of the FTMR used in this trial, approximately 60%, meant that the availability of juice for fermentation was limited [27], which could have delayed modifications of the NH_3_–N, acetic acid, and propionic acid contents until day 15 of ensiling. Interestingly, similarly to this study, a previous study observed that FTMR with a high DM content (500–600 g/kg) was maintained outdoors for more than 4 months with a pH of 4.3 and minimal dry matter loss during the summer [28]. Moreover, the LAB populations in OS-added treatments were greater than in samples exposed to 0% OS FTMR. This could have also inhibited the growth of yeasts and other undesirable organisms [29], thereby contributing to a shift in the microbiota population and their metabolites. Overall, the addition of 11% or 22% OS FTMR increased the fermentation quality, as a larger population of LAB, smaller lactate/acetate ratio, and quicker spoilage yeast death were detected.

Throughout the study, the loss of DM was less than 1%. The fast decrease in the WSC levels in the first 15 days indicates that WSC is the main substrate present in the first fermentation stage. Regarding the protein fraction, no differences in the CP or NPN concentrations were found on days 0 or 60. The NH_3_–N fraction was 6.01% after fermentation, which is lower than that present in normal alfalfa and stylo silage, as reported by Wang et al. (2019) [21]. This indicates that FTMR retained the utilization efficiency of protein, given that the efficiency of rumen microbial-N synthesis is higher when silage is supplemented with protein-N rather than nonprotein-N [30]. Although the mechanism by which proteins in FTMR is degraded during the ensiling process can vary, there is a widespread notion that plant proteases and microbial activities play dominant roles in protein breakdown [31,32]. The dynamics of nutrient degradation and the roles of specific bacteria [28] have been elucidated; however, to date, little is known about the correlations between these chemicals and the bacterial structure at the genus or species levels.

Different types of microorganisms, like enterobacteria, yeasts, and filamentous fungi, compete with LAB to utilize substrates and produce CO_2_ during ensiling, which could cause a dynamic community shift during ensiling [33]. The present study showed that the *Lactobacillus* genus complex was the dominant genus after 60 days of ensiling, while there was a diverse range of undesirable bacteria present in the unfermented TMR. Consistent with our observations, Ni et al. [34] noticed that the most abundant microorganisms in mixed silage were those belonging to *Lactobacillus* genus complex, which reached an abundance level of nearly 90% and might play a critical role in its relatively good fermentation. When organized as dairy starters, the *Lactobacillus* genus complex was shown to play an important role in souring raw milk [35]. Moreover, a higher milk fat concentration was found when a portion of FTMR containing wet corn gluten and corn stover was used to replace alfalfa hay in dairy cow feed [36]. This might be attributed to the presence of ruminal lactate-utilizing bacteria, which can convert lactic acid to propionic acid by secondarily fermenting lactic acid [37]. Samples treated with 0% TMR differed from those treated with 11% and 22% OS FTMR in terms of the relative abundance of species of *Lactobacillus* gnus complex, indicating that the addition of OS shifted the microbial community at the species level on day 60. A lower concentration of homofermentative *L. acetotolerans* was observed in the 11% and 22% OS FTMR samples. Han et al. [38] also reported that *L. acetotolerans* was the only LAB found both in corn FTMR and the ruminant gastrointestinal tract, which is consistent with our observations. Notably, it was previously demonstrated that, in terms of short chain fatty acid profiles, the 22% OS group had the lowest lactic acid/acetic acid in the FTMR. The higher the amount of OS added into the TMR, the more diverse heterofermentative species of *Lactobacillus* genus complex found on day 60. In particular, there were more heterolactic acid bacteria, for example, *L. buchneri, L. brevis*, and *C. versmoldensis,* with the ability to produce less lactic acid and more acetic acid in 22% OS FTMR. In summary, the addition of OS shifted the fermentation pattern from homofermentation to heterofermentation.

The *Lactobacillus* genus complex is often characterized by utilizing fermentable substrates to produce organic acids, leading to a decrease in pH [39,40]. In addition, the presence of *L. coryniformis*, *L. acidipiscis*, and *Lactobacillus* compost bacterium was linked with the NPN concentration, suggested that the species of *Lactobacillus* genus complex may carry out N metabolism. A similar observation by Guo et al. [41] showed that *L. buchneri*, belonging to *Lactobacillus* genus complex, could produce some amino acids in silage. Most likely, *L. coryniformis*, a member of the *Lactobacillus* genus complex, was involved in NPN synthesis. Taxonomy alone cannot be used to define a given microbiome and state its connection to the environment for biological and technical reasons [42], which is one limitation of the present study. To better interpret the function of *Lactobacillus* species of FTMR with more OS, species with close connections to the fermentation of FTMR could indicate a direction for further manipulation of bacterial communities.

## 5. Conclusions

Adding oat silage could improve the fermentation quality of FTMR by providing a lower butyric acid concentration and lactic acid/acetic acid ratio and a larger LAB population. In line with this, stable CP and NPN concentrations were found. Larger relative abundance of heterofermentative LAB, *L. buchneri, L. brevis*, and *C. versmoldensis* were found in samples containing 11% or 22% OS after 60 days of ensiling. Among these, the presence of species of the *Lactobacillus* genus complex, such as *L. coryniformis*, was strongly associated with the FTMR chemical composition, suggesting the potential role of these species in the fermentation profiles and metabolism pathways.

## Figures and Tables

**Table 1:** Ingredients and nutrient levels of the total mixed rations (TMRs).

Items	0% ^1^	11% ^2^	22% ^3^
Ingredients (%DM)			
Oat Silage	0.00	11.00	22.00
Alfalfa hay	14.18	14.18	14.18
Oat hay	4.76	4.76	4.76
Corn silage	22.00	11.00	0.00
Concentrate	59.06	59.06	59.06
Total	100.00	100.00	100.00
Nutrient level (%DM)			
Dry matter	58.69	59.49	62.51
NEL (MJ Kg^−1^)	6.73	6.76	6.76
Crude protein	15.97	15.72	15.90
Water-soluble carbohydrates	11.35	11.64	11.43
Neutral detergent fiber	35.40	33.99	33.74
Acid detergent fiber	20.97	20.75	20.82

0% ^1^, 0% oat silage TMR; 11% ^2^, 11% oat silage TMR; 22% ^3^, 22% oat silage TMR.TMR, total mixed ration; DM, dry matter; NEL, net energy for lactation.

**Table 2:** Fermentation characteristics of fermented total mixed ration (FTMR) with different content of oat silage in ensiling periods.

Item	OS	Days of Ensiling								SEM	*p*-Value		
		0	3	5	7	10	15	30	60		OS	Days	OS × Days
pH	0%	5.38 ^bB^	5.74 ^aA^	5.43 ^b^	5.29 ^bAB^	4.87 ^cB^	4.56 ^dB^	4.42 ^d^	4.49 ^dB^	0.03	0.015	<0.001	0.062
	11%	5.62 ^aA^	5.61 ^aAB^	5.68 ^a^	5.66 ^aA^	5.27 ^bA^	4.84 ^cA^	4.56 ^d^	4.66 ^c^^dA^				
	22%	5.50 ^aAB^	5.32 ^a^^bB^	5.35 ^a^^b^	5.11 ^bB^	4.66 ^cC^	4.66 ^cB^	4.61 ^c^	4.57 ^cAB^				
NH_3_-N	0%	3.78 ^b^^cd^	4.00 ^b^^cd^	3.30 ^c^^d^	2.97 ^d^	4.47 ^b^	4.17 ^b^^c^	3.91 ^b^^cd^	5.48 ^a^	0.14	0.703	<0.001	0.941
(%TN)	11%	3.23 ^c^^d^	3.38 ^c^^d^	2.99 ^d^	4.01 ^b^^cd^	4.51 ^b^^c^	3.15 ^d^	4.91 ^b^	6.35 ^a^				
	22%	2.79 ^bc^	2.23 ^c^	3.57 ^bc^	3.97 ^bc^	3.70 ^bc^	3.98 ^bc^	4.70 ^b^	6.20 ^a^				
Lactic acid	0%	3.23 ^a^^bAB^	2.44 ^b^	2.53 ^b^	2.12 ^b^	2.62 ^bA^	2.33 ^b^	4.81 ^a^	3.42 ^a^^bA^	0.17	0.016	<0.001	0.375
(%DM)	11%	4.95 ^aA^	4.35 ^a^	4.14 ^a^	3.90 ^a^	0.97 ^bB^	2.90 ^ab^	3.80 ^a^	3.11 ^abA^				
	22%	1.53 ^bB^	1.36 ^b^	3.44 ^a^	3.45 ^a^	2.31 ^bcA^	1.55 ^b^	3.63 ^a^	1.98 ^bcB^				
Acetic acid	0%	1.09 ^b^	0.80 ^b^	1.22 ^b^	0.84 ^b^	1.14 ^b^	0.77 ^b^	2.24 ^a^	2.91 ^a^	0.11	0.582	<0.001	0.069
(%DM)	11%	1.11 ^a^^b^	1.45 ^a^^b^	1.00 ^a^^b^	1.18 ^a^^b^	0.38 ^b^	1.52 ^a^^b^	2.04 ^a^	1.47 ^a^^b^				
	22%	0.94 ^b^	1.35 ^b^	1.44 ^b^	1.37 ^b^	1.13 ^b^	0.85 ^b^	2.51 ^a^	2.45 ^a^				
Propionic acid	0%	0.03 ^b^	0.09 ^a^^b^	0.12 ^a^^b^	0.09 ^a^^b^	0.08 ^a^^b^	0.04 ^b^	0.14 ^a^^b^	0.20 ^a^	0.03	0.068	<0.001	0.156
(%DM)	11%	0.07 ^b^^c^	0.13 ^a^^bc^	0.03 ^c^	0.04 ^c^	0.10 ^b^^c^	0.19 ^bc^	0.37 ^a^	0.33 ^a^^b^				
	22%	0.06 ^b^	0.12 ^b^	0.11 ^b^	0.07 ^b^	0.06 ^b^	0.05 ^b^	0.40 ^a^	0.47 ^a^				
Butyric acid	0%	0.11	0.60 ^A^	0.79 ^A^	0.38 ^A^	0.10	0.12	0.36	0.44 ^A^	0.04	0.014	0.113	0.127
(%DM)	11%	0.14	0.14 ^B^	0.31 ^B^	0.24 ^B^	0.08	0.14	0.32	0.09 ^B^				
	22%	0.28	0.42 ^A^	0.37 ^B^	0.12 ^B^	0.10	0.12	0.12	0.07 ^B^				

^a–d^ Means within the same row with different superscripts differ significantly among ensiling days (one-way ANOVA, *p* < 0.05, Tukey’s post hoc test). ^A–C^ Means within the same column with different superscripts differ significantly among OS treatments (one-way ANOVA, *p* < 0.05, Tukey’s post hoc test). 0%, 0% oat silage; 11%, 11% oat silage; 22%, 22% oat silage. OS, oat silage content; FTMR, fermented total mixed ration; SEM, standard error of the mean; TN, total nitrogen; DM, dry matter.

**Table 3:** Chemical composition of FTMR with different contents of oat silage in ensiling periods.

Item	OS	Days of Ensiling								SEM	*p*-Value		
		0	3	5	7	10	15	30	60		OS	Days	OS × Days
DML	0%	0.00 ^c^	0.00 ^c^	0.09 ^bc^	0.18 ^bc^	0.27 ^abc^	0.37 ^abc^	0.55 ^ab^	0.74 ^a^	0.02	0.163	<0.001	0.232
(%DM)	11%	0.00 ^d^	0.09 ^cd^	0.28 ^bcd^	0.19 ^bcd^	0.18 ^bcd^	0.55 ^ab^	0.46 ^abc^	0.74 ^a^				
	22%	0.00 ^d^	0.18 ^cd^	0.18 ^cd^	0.28 ^bcd^	0.55 ^abc^	0.55 ^abc^	0.64 ^ab^	0.83 ^a^				
NDF	0%	31.77 ^c^	33.36 ^abc^	32.43 ^abcB^	32.12 ^bc^	34.55 ^a^	33.87 ^abc^	32.87 ^abc^	34.19 ^ab^	0.22	< 0.001	<0.001	0.681
(%DM)	11%	33.99 ^bc^	33.34 ^c^	37.95 ^aA^	32.73 ^c^	36.34 ^abc^	37.12 ^ab^	34.23 ^bc^	33.55 ^bc^				
	22%	33.17 ^cd^	33.87 ^bcd^	33.98 ^dB^	33.35 ^bcd^	36.24 ^ab^	36.62 ^a^	34.05 ^bcd^	34.82 ^abc^				
ADF	0%	20.98 ^bc^	21.26 ^bc^	21.36 ^bc^	21.69 ^bc^	25.66 ^a^	20.41 ^bc^	18.95 ^c^	23.14 ^ab^	0.25	0.123	0.022	0.891
(%DM)	11%	20.61 ^c^	21.47 ^abc^	25.27 ^a^	22.35 ^abc^	24.82 ^ab^	22.57 ^abc^	20.94 ^bc^	22.25 ^abc^				
	22%	20.82 ^b^	23.45 ^a^	21.82 ^ab^	23.86 ^a^	20.99 ^ab^	23.58 ^a^	19.74 ^b^	23.89 ^a^				
CP	0%	15.98 ^ab^	16.08 ^ab^	15.76 ^ab^	15.95 ^ab^	15.15 ^b^	15.72 ^ab^	16.51 ^a^	15.82 ^ab^	0.10	0.706	<0.001	0.068
(%DM)	11%	15.43 ^c^	16.84 ^a^	15.67 ^bc^	15.86 ^bc^	16.36 ^ab^	16.17 ^abc^	15.67 ^bc^	15.94 ^bc^				
	22%	15.90 ^ab^	15.58 ^ab^	15.42 ^ab^	15.68 ^ab^	15.03 ^b^	15.73 ^ab^	16.37 ^a^	15.51 ^ab^				
WSC	0%	10.66 ^b^	12.21 ^aA^	11.62 ^abA^	11.69 ^abA^	8.92 ^c^	5.18 ^d^	3.71 ^e^	2.57 ^e^	0.24	< 0.001	<0.001	0.093
(%DM)	11%	11.43 ^a^	9.00 ^abB^	8.48 ^abB^	4.75 ^bcB^	7.02 ^abc^	6.42 ^bc^	3.08 ^c^	2.84 ^c^				
	22%	11.64 ^a^	9.08 ^bcB^	8.51 ^cB^	9.91 ^bA^	7.11 ^d^	4.84 ^e^	3.29 ^f^	2.28 ^f^				
NPN	0%	31.26 ^c^	28.72 ^abc^	30.91 ^bc^	28.72 ^bc^	26.26 ^bc^	31.40 ^ab^	37.14 ^ab^	35.54 ^a^	0.60	0.392	<0.001	0.122
(%CP)	11%	27.72 ^c^	32.35 ^c^	31.06 ^bc^	29.53 ^bc^	28.25 ^c^	33.69 ^ab^	33.45 ^a^	37.58 ^a^				
	22%	27.32 ^de^	26.20 ^de^	30.24 ^cd^	28.77 ^de^	24.30 ^e^	35.49 ^bc^	39.17 ^ab^	39.89 ^a^				

^a–f^ Means within the same row with different superscripts differ significantly among ensiling days (one-way ANOVA, *p* < 0.05, Tukey’s post hoc test). ^A–C^ Means within the same column with different superscripts differ significantly among OS treatments (one-way ANOVA, *p* < 0.05, Tukey’s post hoc test). 0%, 0% oat silage; 11%, 11% oat silage; 22%, 22% oat silage. OS, oat silage content; FTMR, fermented total mixed ration; DML, dry matter loss during the ensiling period; FM, fresh matter; NDF, neutral detergent fiber; ADF, acid detergent fiber; CP, crude protein; WSC, water-soluble carbohydrates; NPN, non-protein nitrogen; SEM, standard error of the mean.
